# LiteMS-YOLO: a lightweight framework for small target detection in complex wheat field environments

**DOI:** 10.3389/fpls.2026.1851297

**Published:** 2026-06-05

**Authors:** Hongliang Ma, Maoxing Song, Mengying Yang, Tong Li, Xuefei Wang, Mochu Wang, Xiaoxue Zhao, Cheng Peng, Huina Huang, Peng Wu, Qing Lu, Zhihui Wu

**Affiliations:** 1 Tangshan Academy of Agricultural Sciences, Tangshan, China; 2 Guye District Market Supervision Administration of Tangshan, Tangshan, China; 3 College of Animal Science and Technology, Hebei Agricultural University, Baoding, China

**Keywords:** lightweight object detection, multi-scale feature extraction, small object detection, wheat spike detection, YOLO-based model

## Abstract

Wheat spike detection is essential for yield estimation in precision agriculture, yet it remains challenging due to the small size of targets, dense distribution, and complex field environments. In this study, we propose LiteMS-YOLO, a lightweight object detection framework based on YOLO26n. The model integrates a Feature Complementary Mapping (FCM) module to enhance spatial-semantic feature interaction and a Multi-Kernel Perception (MKP) unit to improve multi-scale feature representation. In addition, targeted redundancy reduction strategies are introduced to significantly lower model complexity. Experiments are conducted on a combined dataset comprising the public Global Wheat Head Detection (GWHD) dataset and 100 field images collected by the Tangshan Academy of Agricultural Sciences, with a total of 6,378 high-resolution images and over 44,000 annotated wheat spikes. LiteMS-YOLO achieves a mAP50 of 92.28% and a mAP50–95 of 52.56%, while using only 0.627 million parameters. Compared with YOLO26n and YOLOv8n, the proposed method reduces parameters by approximately 75% and 79%, respectively, while maintaining competitive accuracy. These results demonstrate that LiteMS-YOLO strikes an excellent balance between detection accuracy and efficiency, making it well-suited for real-time deployment in resource-constrained agricultural scenarios.

## 1 Introduction

Wheat is one of the most important staple crops worldwide and plays a critical role in global food security. Accurate estimation of wheat yield is essential for crop management, breeding programs, and precision agriculture. Among various agronomic traits, the number and spatial distribution of wheat spikes are key indicators for yield estimation and phenotypic analysis (Hasan et al., 2018; Xiong et al., 2019). The availability of large-scale annotated datasets, such as the Global Wheat Head Detection (GWHD) (David et al., 2020) dataset, has significantly promoted the development of automated detection methods.

Traditional manual counting methods are labor-intensive, time-consuming, and prone to human error, making them unsuitable for large-scale agricultural applications. With the rapid advancement of computer vision, deep learning-based approaches have been widely adopted for plant phenotyping and agricultural monitoring. Early studies mainly relied on convolutional neural networks (CNNs) to detect wheat spikes, demonstrating promising results compared to traditional image processing methods.

Object detection frameworks have further improved the performance of wheat spike detection. Two-stage detectors, such as Faster R-CNN, achieve high detection accuracy but suffer from high computational complexity and limited real-time capability (Ren et al., 2017). In contrast, single-stage detectors represented by the YOLO (You Only Look Once) (Redmon et al., 2016) series provide a better trade-off between speed and accuracy, making them more suitable for real-time agricultural applications (Bochkovskiy et al., 2020; Terven and Cordova-Esparza, 2023). As a result, numerous studies have proposed improved YOLO-based models for wheat spike detection by incorporating attention mechanisms, feature pyramid networks, and lightweight structures (Zang et al., 2022; Guan et al., 2024; Qiu et al., 2025; Wang et al., 2025a; Wu et al., 2025).

Despite these advances, wheat spike detection in real-world field environments remains highly challenging. First, wheat spikes are typically small objects, especially in UAV imagery, and are easily lost during deep feature extraction due to repeated downsampling operations (Kong et al., 2024). Second, spikes are densely distributed and often occluded, resulting in missed detections and inaccurate localization (Wen et al., 2024). Third, variations in illumination, background complexity, and growth stages further degrade detection robustness (Zhaosheng et al., 2022; Wang et al., 2024).

To address these challenges, recent studies have focused on enhancing multi-scale feature representation and feature fusion strategies. Feature pyramid networks (FPN) (Lin et al., 2017; Liu et al., 2024) and path aggregation networks (PANet) (Liu et al., 2018) have been widely adopted to improve multi-scale feature learning. Additionally, attention mechanisms such as CBAM (Cheng et al., 2024) and ECA-Net (Wang et al., 2020; Yang et al., 2022) have been introduced to enhance feature representation by emphasizing important spatial and channel information. More recently, transformer-based detection frameworks, such as DETR (Hoanh and Pham, 2024; Li et al., 2024) and its variants, have shown strong capability in modeling global contextual information.

Among recent advances, FBRT-YOLO (Xiao et al., 2025) has demonstrated promising performance in aerial image detection by addressing feature imbalance and enhancing multi-scale perception. Specifically, it introduces the Feature Complementary Mapping (FCM) module to improve the interaction between spatial and semantic features, as well as the Multi-Kernel Perception (MKP) unit to capture multi-scale contextual information efficiently. These designs provide valuable insights for improving small-object detection in complex scenarios.

However, directly applying such designs to wheat spike detection remains insufficient. Wheat spikes exhibit unique characteristics, including extreme density, severe occlusion, and subtle differences from background regions, which impose stricter requirements on spatial information preservation and lightweight model design. Moreover, existing approaches often fail to fully balance detection accuracy and computational efficiency, limiting their deployment in real-time agricultural applications.

To address these limitations, this study proposes a novel lightweight detection framework termed LiteMS-YOLO, built upon the YOLO26 (Hidayatullah and Tubagus, 2026) architecture. YOLO26 is selected due to its end-to-end NMS-free design and superior small-object detection capability. Inspired by FBRT-YOLO, the proposed model incorporates an improved Feature Complementary Mapping (FCM) module to enhance spatial-semantic feature interaction, and a redesigned Multi-Kernel Perception (MKP) unit to strengthen multi-scale representation while reducing computational overhead. Furthermore, targeted redundancy reduction strategies are introduced to significantly decrease model parameters without sacrificing detection accuracy.

Compared with FBRT-YOLO, the proposed LiteMS-YOLO introduces task-oriented structural modifications at both the feature interaction level and the multi-scale perception level, specifically for densely distributed small wheat spikes.

### 1.1 Differences in FCM design

In FBRT-YOLO, the FCM module mainly focuses on alleviating spatial–semantic information imbalance by transferring shallow spatial cues into deeper semantic layers through a symmetric split-transform-aggregation strategy. However, it does not explicitly consider the progressive degradation of spatial information in deeper layers.

In contrast, the FCM in LiteMS-YOLO is redesigned with a depth-aware channel allocation mechanism. Specifically, larger channel ratios are assigned to semantic branches in shallow layers, while deeper layers allocate more channels to spatial information preservation. This asymmetric design explicitly compensates for the spatial information loss caused by repeated downsampling, which is particularly critical for densely distributed small targets such as wheat spikes. As a result, the proposed FCM is not only a feature fusion module but also a spatial information rebalancing mechanism across network depth.

### 1.2 Differences in MKP structure

In FBRT-YOLO, MKP adopts a multi-kernel perception strategy primarily to enhance scale diversity, where multiple receptive fields are introduced to improve detection across different object sizes. However, its design mainly focuses on general multi-scale representation rather than efficiency-oriented integration.

In LiteMS-YOLO, the MKP unit is further restructured into a sequential multi-kernel aggregation framework. Instead of loosely enhancing multi-scale features, the proposed MKP progressively expands the receptive field through cascaded depthwise convolutions (3×3, 5×5, 7×7), interleaved with point-wise convolutions. This design ensures continuous receptive field growth while maintaining spatial coherence, which is more suitable for small and overlapping targets. Moreover, the MKP unit simultaneously replaces the final downsampling layer, unifying feature extraction and resolution reduction into a single operation, thereby reducing redundant computation.

### 1.3 Additional architectural optimization beyond FBRT-YOLO

Unlike FBRT-YOLO, which mainly introduces FCM and MKP as plug-in modules, LiteMS-YOLO further incorporates a targeted redundancy reduction strategy. Specifically, conventional downsampling blocks are decoupled into lightweight group convolution and point-wise convolution, significantly reducing parameter redundancy while preserving feature representation capability. This design is particularly important for UAV edge deployment scenarios, where computational resources are strictly limited.

Overall, while FBRT-YOLO focuses on improving general small-object perception in aerial images, LiteMS-YOLO introduces depth-aware feature rebalancing and computation-aware structural redesign, making it more suitable for dense, occluded, and small-scale agricultural targets.

## 2 Related works

The integration of deep learning into precision agriculture has significantly advanced automated crop monitoring and phenotyping. Convolutional neural networks (CNNs) have demonstrated strong capabilities in feature extraction from high-resolution images, enabling automated tasks such as crop disease identification, pest detection, and weed density estimation. These developments have laid the foundation for high-throughput phenotypic analysis of key agronomic traits, including wheat spike detection, which is central to yield estimation and breeding programs.

Early approaches to wheat spike detection relied primarily on traditional image processing techniques, such as color thresholding and morphological operations. However, these methods exhibit limited robustness under varying illumination, occlusion, and overlapping spike conditions commonly encountered in field environments. The introduction of deep learning-based object detection frameworks, particularly two-stage detectors like Faster R-CNN (Liu et al., 2026) and single-stage detectors such as the YOLO series, marked a significant performance breakthrough. The release of large-scale annotated datasets, including the Global Wheat Head Detection (GWHD) dataset, further accelerated progress by enabling systematic benchmarking of detection algorithms across diverse geographic and environmental conditions (Khazmi and Lachiri, 2024).

In recent years, the YOLO series has undergone rapid evolution, progressively improving detection accuracy and inference speed. YOLOv5 and YOLOv8 introduced anchor-free detection spikes and enhanced neck architectures, substantially improving small object recall (Fang and Yang, 2024). YOLOv9 incorporated Programmable Gradient Information (PGI) and Generalized Efficient Layer Aggregation Network (GELAN) to improve gradient flow and feature reuse. YOLO11 further optimized the architecture with enhanced C3k2 modules and depth-wise separable convolutions, achieving competitive performance with reduced computational costs (Lei et al., 2025; Zhang et al., 2025). These advancements provide a strong backbone for task-specific adaptations in agricultural scenarios, where efficiency and accuracy must be jointly optimized for edge deployment.

Lightweight network design has emerged as a critical research direction for enabling real-time inference on resource-constrained platforms. Techniques such as depthwise separable convolution, channel pruning, knowledge distillation, and neural architecture search have been widely adopted to reduce model size and computational overhead (Liang et al., 2025). In particular, structural reparameterization methods, exemplified by RepVGG and its derivatives, offer an effective way to decouple training-time multi-branch architectures from inference-time single-branch representations, achieving a favorable accuracy-efficiency trade-off without additional inference latency. These lightweight strategies are especially relevant in agricultural field applications where edge devices and UAV onboard processors impose strict constraints on model complexity.

Multi-scale feature representation is a fundamental challenge in detecting small and densely packed objects such as wheat spikes. Feature Pyramid Network (FPN) and its variants, including Path Aggregation Network (PANNet) and BiFPN, have been widely employed to fuse features across different resolution levels, improving detection performance for both small and large objects simultaneously (Zou et al., 2023; Wang et al., 2025b). More recently, attention mechanisms including Squeeze-and-Excitation (SE), Convolutional Block Attention Module (CBAM), and Deformable Convolutional Networks have been incorporated into detection backbones and necks to enhance spatial and channel-wise feature selectivity (Zou et al., 2023). These modules allow detectors to focus on informative regions while suppressing background noise, which is particularly beneficial in cluttered wheat field scenes where spikes exhibit high visual similarity and irregular spatial distributions.

The advent of Transformer-based architectures has opened new avenues for wheat detection. Detection Transformer (DETR) and its improved variants, such as RT-DETR and WH-DETR, leverage global self-attention mechanisms to capture long-range spatial dependencies, demonstrating strong performance in complex agricultural backgrounds. However, Transformer-based models typically demand substantially higher computational resources compared to CNN-based counterparts, making their deployment on UAV edge platforms challenging. This gap underscores the continued relevance of efficient convolutional architectures for practical precision agriculture applications, particularly when real-time processing under hardware constraints is required.

Despite these advances, several key challenges remain in wheat spike detection from ground-level and near-ground imagery. Existing methods often struggle with dense target arrangements, mutual occlusion among adjacent spikes, and high intra-class appearance variability across different growth stages, viewing angles, and lighting conditions. Furthermore, most current detectors are not explicitly optimized for small-target detection in complex field environments with cluttered backgrounds. The proposed LiteMS-YOLO framework addresses these limitations by integrating a Feature Complementary Mapping (FCM) module for enhanced spatial-semantic feature interaction, a Multi-Kernel Perception (MKP) unit for robust multi-scale representation, and targeted redundancy reduction strategies to maintain competitive accuracy under strict computational budgets.

## 3 Methodology

### 3.1 Overview of LiteMS-YOLO

**Figure 1:**
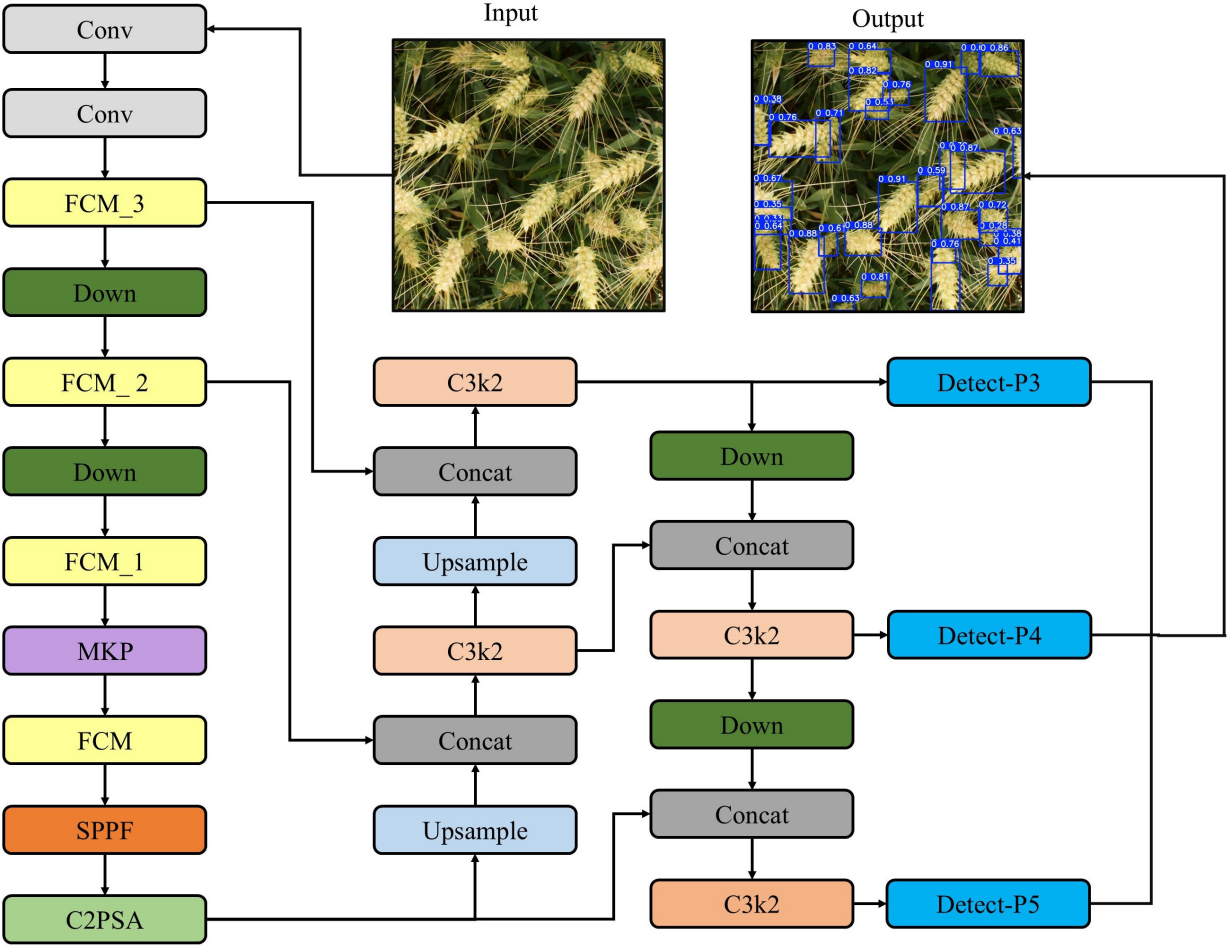
Overall architecture of LiteMS-YOLO.

LiteMS-YOLO is built upon the YOLO26 architecture [27], a state-of-the-art real-time object detector featuring end-to-end NMS-free inference, dual label assignment, and small-target-aware optimization. The overall architecture of LiteMS-YOLO is illustrated in **Figure 1**. The model follows the standard YOLO pipeline with three main components: backbone, neck, and detection head. The key innovations lie in the integration of two lightweight modules: Feature Complementary Mapping (FCM) and Multi-Kernel Perception (MKP).

**Figure 2:**
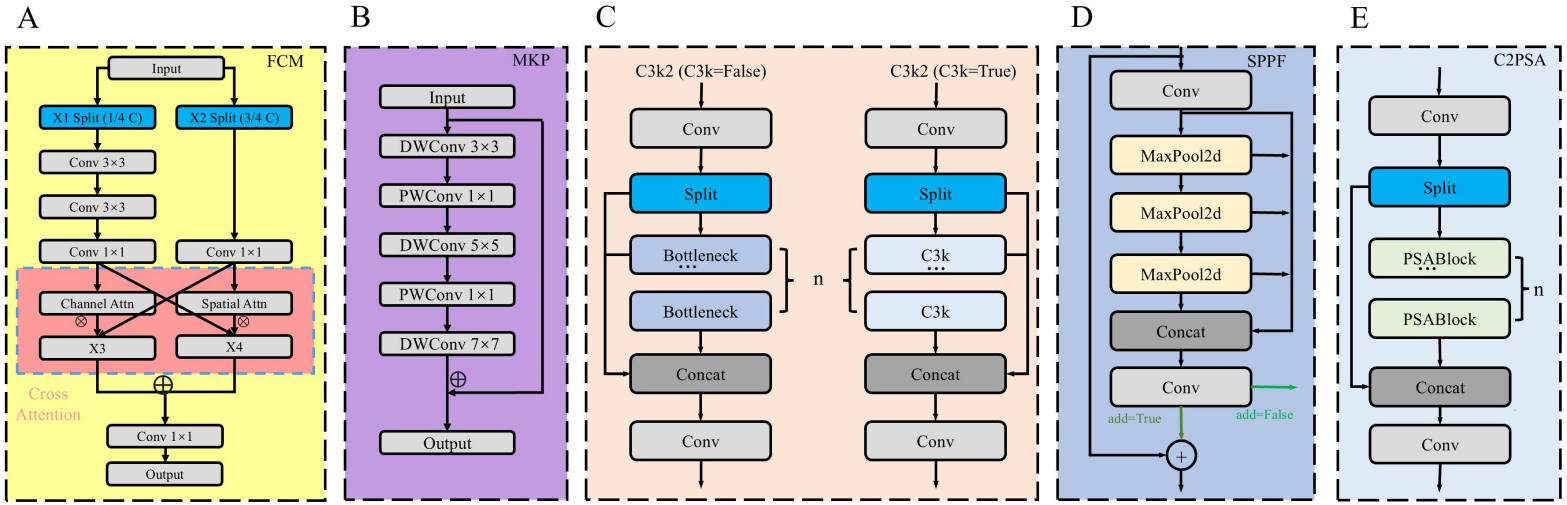
Key modules of LiteMS-YOLO. **(A)** FCM Module. **(B)** MKP Module. **(C)** C3k2 Module. **(D)** SPPF Module. **(E)** C2PSA Module.

The backbone network begins with two convolutional layers (kernel size 3, stride 2) that progressively reduce spatial resolution. Subsequently, the FCM module is embedded at multiple stages of the backbone to propagate shallow spatial information into deeper layers, enhancing feature alignment for small target localization. The MKP unit replaces the final downsampling layer, employing multi-scale depthwise convolutions to enhance perception across varying scales while reducing computational redundancy. The neck incorporates upsampling and concatenation operations to fuse features at multiple scales, followed by C3k2 blocks that further refine feature representations. In addition, the SPPF module is employed to enlarge the receptive field and enhance multi-scale contextual information, while the C2PSA module further improves feature representation by strengthening spatial attention and channel interaction. The detailed structures of the C3k2, SPPF, and C2PSA modules are illustrated in **Figures 2C–E**, respectively. Finally, the detection head generates predictions for small, medium, and large objects through three detection branches. 

### 3.2 Feature Complementary Mapping module

The Feature Complementary Mapping (FCM) module addresses the critical challenge of information imbalance between shallow spatial features and deep semantic representations in convolutional networks. As shown in **Figure 2A**, FCM employs a four-stage strategy: channel split, orientation transformation, complementary mapping, and feature aggregation.

#### 3.2.1 Channel Split

Given an input feature map X_input_∈R^(C×H×W)^, the channels are split into two branches with a ratio α and (1−α), where α∈[0,1] controls the proportion of channel information. This split can be formulated as Equation 1:

(1) $$X_{1},X_{2}=Split\left(X_{input}\right)$$

where X_1_∈R ^(αC×H×W)^ and X_2_∈R^((1-α)C×H×W).^

#### 3.2.2 Orientation transformation

The two branches undergo different transformations to capture complementary information. X_1_ is processed through a 3×3 standard convolution to extract rich channel information, denoted as X_C_. X_2_ is processed through point-wise (1×1) convolution to preserve shallow spatial information, denoted as

X_S_. This transformation can be expressed as Equation 2:

(2) $$\left(X_{C},X_{S}\right)=\varphi_{1}\left(X_{1},X_{2}\right)$$

where ϕ_1_ represents the mapping function that transforms spatial and semantic information.

#### 3.2.3 Complementary mapping

To achieve efficient feature matching, complementary mapping is performed between the two branches. Channel interaction is applied to X_C_ using depthwise convolution followed by global average pooling and sigmoid activation to generate channel attention weights ω_1_ shown as Equations 3, 4.

(3) $$X_{D}=DepthwiseConv\left(X_{C}\right)$$

(4) $$\omega_{1}=\sigma \left(\frac{1}{H\times W}\sum_{i,j}X_{D}\left(i,j\right)\right)$$

where σ denotes the sigmoid activation function. Similarly, spatial interaction is applied to X_S_ using a 1×1 spatial convolution with batch normalization and sigmoid to generate spatial attention weights ω_2_ shown as Equation 5.

(5) $$\omega_{2}=\sigma \left(BN\left(Conv_{\left(1\times 1\right)\left(X_{S}\right)}\right)\right)$$

#### 3.2.4 Feature aggregation

The final output

X_FCM_ is obtained by aggregating the complementary features shown as Equation 6:

(6) $$X_{FCM}=\left(X_{C}⊗\omega_{2}\right)⊕\left(X_{S}⊗\omega_{1}\right)$$

where ⊗ denotes element-wise multiplication and ⊕ denotes element-wise addition. This aggregation enables the network to integrate both rich semantic information from channel interactions and precise spatial location information from spatial interactions.

The split ratio αis a critical hyperparameter that balances spatial and channel information propagation. Based on empirical analysis, we adopt a configuration of α = [0.75,0.75,0.25,0.25] for the four backbone stages, retaining more spatial location information in deeper layers where semantic information dominates.

The rationale behind assigning larger α values in shallow layers and smaller values in deeper layers is based on the characteristics of small object detection. As network depth increases, semantic information becomes dominant while spatial resolution decreases. However, accurate localization of small and densely distributed wheat spikes relies heavily on spatial details. Therefore, allocating more channels to preserve spatial information in deeper layers helps compensate for the loss caused by repeated downsampling, improving detection accuracy.

### 3.3 Multi-Kernel Perception unit

The Multi-Kernel Perception (MKP) unit is designed to enhance multi-scale target perception while maintaining computational efficiency. As illustrated in **Figure 2B**, MKP sequentially applies depthwise convolutions with varying kernel sizes, interleaved with point-wise convolutions to integrate spatial information across scales.

Given an input feature X, MKP processes it through a cascade of operations shown as Equation 7:

(7) $$X^{\prime }=T_{\left(2k+1\right)}\left(A\left(\ldots A\left(T_{k\left(X\right)}\right)\ldots \right)\right)$$

where T_k_ represents depthwise convolution with kernel size k, and A represents point-wise convolution transformation. In our implementation, we employ kernel sizes of 3, 5, and 7, with point-wise convolutions interleaved between them. This sequential design allows the network to progressively expand its receptive field while maintaining fine-grained detail through the point-wise convolutions that preserve channel interactions.

The use of depthwise convolitions significantly reduces computational complexity compared to standard convolutions. For a convolution with kernel size k, input channels C_1_, and output channels C_2_, the parameter count is shown as Equation 8:

(8) $$P_{depthwise}=k\times k\times C_{1}$$

whereas standard convolution would require k×k×C_1_×C_2_ parameters. This efficiency enables the incorporation of multiple kernel scales without substantially increasing model size.

MKP replaces the final downsampling layer in the backbone network, simultaneously performing spatial downsampling while extracting multi-scale features. This design choice eliminates redundant computation by unifying downsampling and multi-scale feature extraction into a single operation.

### 3.4 Targeted reduction of redundancy

In addition to the FCM and MKP modules, we implement targeted reductions in architectural redundancy to further improve efficiency. Conventional downsampling blocks typically expand channels before depthwise convolution, leading to spatial information loss and increased computation. Instead, we decouple this process by first applying group convolution for spatial downsampling, followed by point-wise convolution for channel expansion.

For a downsampling operation with input channels C_1_ and output channels C_2_ = 2C_1_, standard convolution is shown as Equation 9:

(9) $$P_{std}=3\times 3\times C_{1}\times C_{2}=18C_{1}^{2}$$

Our decoupled approach with group convolution (g groups) and point-wise convolution is shown as Equation 10:

(10) $$P_{ours}=3\times 3\times C_{1}\times \frac{C_{1}}{g}+1\times 1\times C_{1}\times C_{2}=\frac{9C_{1}^{2}}{g}+2C_{1}^{2}$$

With g = *C*_1_ (depthwise convolution), the parameter count reduces to $9C_{1}^{2}+2C_{1}^{2}$, representing substantial savings, particularly for large channel dimensions.

### 3.5 Model configurations

LiteMS-YOLO follows the scaling conventions of YOLO26, with depth_multiple, width_multiple, and max_channels controlling model capacity. The standard configuration employs a width multiplier of 0.25 and depth multiplier of 0.50, resulting in a lightweight model with approximately 0.627 million parameters. The model outputs predictions at three scales corresponding to downsampling factors of 8, 16, and 32, enabling effective detection of small, medium, and large wheat spikes.

## 4 Experiments

### 4.1 Dataset and evaluation metrics

#### 4.1.1 Dataset

Experiments are conducted on a combined dataset consisting of the publicly available Global Wheat Head Detection (GWHD) dataset and a self-collected wheat spike image dataset provided by the Tangshan Academy of Agricultural Sciences. The dataset contains 6378 high-resolution RGB images with over 44000 annotated wheat spikes. In addition, 100 field images were collected by the Tangshan Academy of Agricultural Sciences under real-world agricultural conditions, covering variations in illumination, growth stages, and complex backgrounds.

The combined dataset improves the diversity and robustness of the training data. Specifically, the GWHD2021 subset comprises high-resolution RGB images captured from 11 countries using various ground-based platforms. According to the dataset guidelines, the acquisition height ranged from 1.8 m to 3.0 m, with Ground Sampling Distance (GSD) varying between 0.10 mm and 0.62 mm across different sub-datasets. All images were uniformly resized to 1024×1024 patches. All images were uniformly annotated and preprocessed, and the dataset was split into training (80%) and validation (20%) sets for experimental evaluation.

#### 4.1.2 Evaluation metrics

The following metrics are used to evaluate detection performance:

Precision: The ratio of true positive detections to all positive detections.

Recall: The ratio of true positive detections to all ground truth objects.

mAP50: Mean Average Precision computed at IoU threshold of 0.5.

mAP50-95: Mean Average Precision averaged over IoU thresholds from 0.5 to 0.95 with step 0.05.

Model efficiency is evaluated using:

Parameters: Total number of trainable parameters (in millions).

GFLOPs: Giga floating-point operations per inference.

Inference Time: Inference time is measured on an NVIDIA RTX 4090 GPU with an input size of 640×640, averaged over 100 runs.

### 4.2 Implementation details

All experiments are conducted using the Ultralytics YOLO framework. The model is trained for 300 epochs using stochastic gradient descent (SGD) optimizer with momentum 0.937 and weight decay 0.0005. The initial learning rate is set to 0.01, with a batch size of 16. Input images are resized to 640 × 640 pixels with mosaic and mixup augmentations applied during training. Experiments are performed on an NVIDIA GeForce RTX 4090 GPU. All compared methods were trained under the same experimental settings for fair comparison. To ensure the reliability of the results, each experiment was repeated three times, and the mean and standard deviation are reported.

The FCM module is inserted at four stages of the backbone, with split ratios α = [0.75,0.75,0.25,0.25] determined through ablation studies. The MKP unit replaces the final downsampling layer, employing kernel sizes of 3, 5, and 7 with point-wise convolutions interleaved.

### 4.3 Comparison with state-of-the-art methods

As shown in **Table 1**, LiteMS-YOLO achieves a mAP50 of 92.28% and a mAP50–95 of 52.56%, demonstrating competitive performance among all compared methods.

**Table 1:** Comparison with state-of-the-art wheat spike detection methods.

Model	Parameters (M)	GFLOPs	Inference time (ms)	Precision (%)	Recall (%)	mAP50 (%)	mAP50-95 (%)	Efficiency score*
YOLOv8n	3.016	8.7	2.3	92.12	82.97	92.54	50.03	30.68
YOLO26n	2.541	22.5	3.1	90.42	84.03	91.87	51.06	36.16
RT-DETR-R18	6.213	136.0	9.8	91.80	83.51	91.71	51.14	14.72
FEWheat-YOLO	7.218	25.3	4.5	91.20	82.10	92.10	52.30	12.65
LGWheatNet	5.620	24.1	4.2	91.83	82.70	91.80	51.90	16.39
LiteMS-YOLO	0.627	22.9	2.6	92.23	84.87	92.28	52.56	147.17

* Efficiency Score = mAP50/Parameters (M).

Notably, LiteMS-YOLO requires only 0.627 million parameters, representing approximately a 75.3% reduction compared to YOLO26n (2.541M parameters) and a 79.2% reduction compared to YOLOv8n (3.016M parameters). Despite the substantial reduction in model size, LiteMS-YOLO maintains comparable detection accuracy, with mAP50 only 0.26% lower than YOLOv8n and 0.41% higher than YOLO26n.

Compared with specialized wheat spike detection models, LiteMS-YOLO achieves a superior balance between accuracy and efficiency. FEWheat-YOLO achieves a slightly lower mAP50 of 92.10% while requiring significantly more parameters, and LGWheatNet also exhibits lower accuracy with higher model complexity. These results highlight the effectiveness of LiteMS-YOLO in achieving an optimal trade-off between detection performance and computational cost.

### 4.4 Ablation studies

As shown in **Table 2**, when both FCM and MKP are integrated with redundancy reduction, the final LiteMS-YOLO model achieves the best performance, reaching a mAP50 of 92.28% and a mAP50–95 of 52.56%. Meanwhile, the number of parameters is significantly reduced from 2.504M in the baseline model to 0.627M.

**Table 2:** Ablation study of LiteMS-YOLO components.

Model	FCM	MKP	RR	Params (M)	mAP50 (%)	mAP50-95 (%)
Baseline	×	×	×	2.504	91.87	51.06
+ RR	×	×	✓	0.604	91.54	50.87
+ FCM	✓	×	✓	0.610	92.05	51.38
+ MKP	×	✓	✓	0.623	92.14	51.45
LiteMS-YOLO	✓	✓	✓	0.627	92.28	52.56

RR denotes redundancy reduction operations.

These results demonstrate that the combination of FCM and MKP modules effectively enhances detection performance, while redundancy reduction plays a critical role in minimizing model complexity without sacrificing accuracy.

### 4.5 Analysis of FCM split ratio

**Table 3** presents an analysis of the split ratio α in the FCM module across the four backbone stages. The split ratio determines the proportion of channels allocated to spatial information preservation versus semantic feature extraction.

**Table 3:** Effect of FCM channel split ratio (α) on detection performance.

Split ratio (α1, α2, α3, α4)	Params (M)	mAP50 (%)	mAP50-95 (%)
(0.50, 0.50, 0.50, 0.50)	0.642	91.18	51.06
(0.25, 0.25, 0.25, 0.25)	0.596	91.22	51.10
(0.75, 0.75, 0.75, 0.75)	0.688	91.39	51.20
(0.75, 0.75, 0.25, 0.25)	0.627	92.28	52.56
(0.50, 0.50, 0.25, 0.25)	0.619	91.35	51.45

The optimal configuration is (0.75, 0.75, 0.25, 0.25), which achieves the best detection performance with a mAP50 of 92.28% and a mAP50–95 of 52.56%, while maintaining a moderate parameter count of 0.627M. This configuration is therefore adopted in the final LiteMS-YOLO model.

### 4.6 Analysis of MKP kernel configurations

**Table 4** explores the effect of different kernel configurations in the MKP unit. Various combinations of kernel sizes are evaluated to determine the optimal multi-scale perception strategy.

**Table 4:** Effect of MKP kernel size configurations on detection performance.

Kernel sizes	Params (M)	GFLOPs	Inference time (ms)	mAP50 (%)	mAP50-95 (%)
(3, 3, 3)	0.622	22.7	2.5	91.30	51.38
(5, 5, 5)	0.628	22.8	2.6	91.35	51.40
(7, 7, 7)	0.634	23.1	2.7	91.39	51.42
(3, 5, 7)	0.627	22.9	2.6	92.28	52.56

The configuration with kernel sizes (3, 5, 7) achieves the best performance, with a mAP50 of 92.28% and a mAP50–95 of 52.56%, while maintaining comparable computational cost (22.9 GFLOPs and 2.6 ms inference time). This result demonstrates that combining multiple kernel sizes effectively enhances multi-scale feature representation.

### 4.7 Qualitative analysis

**Figure 3:**
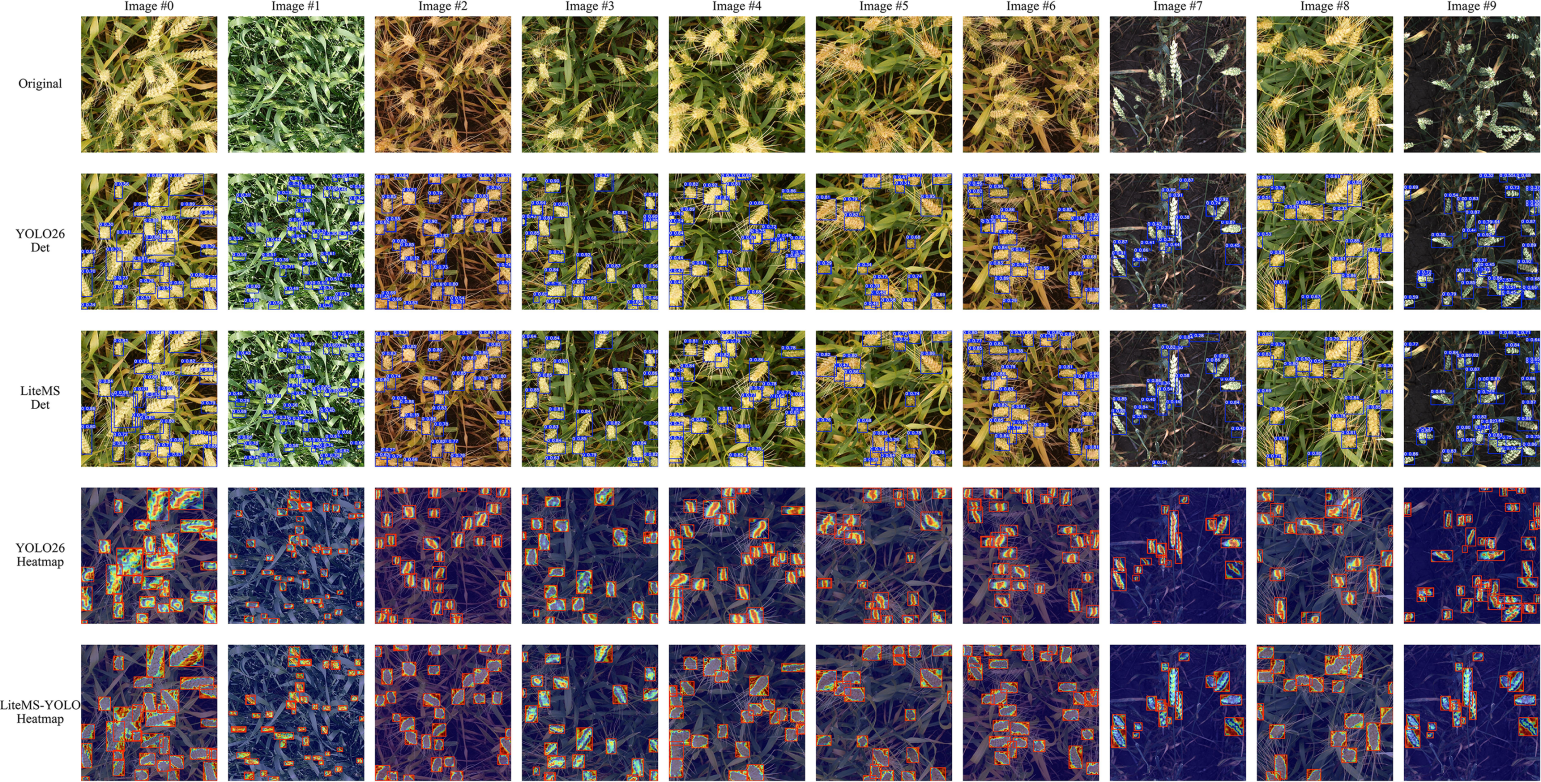
Comprehensive visualization of wheat spike detection and feature maps. First row: Original wheat field images. Second row: Detection results using the baseline YOLO26 model. Third row: Detection results using the proposed LiteMS-YOLO model. Fourth row: Feature map heatmaps from YOLO26 highlighting activation regions. Fifth row: Feature map heatmaps from LiteMS-YOLO demonstrating stronger focus on wheat spikes and reduced background noise.

The first three rows in **Figure 3** presents qualitative detection results comparing baseline YOLO26n with LiteMS-YOLO on representative wheat field images. The visualized results demonstrate LiteMS-YOLO’s superior performance in challenging scenarios:

#### 4.7.1 Dense spike scenarios

In regions with high spike density, LiteMS-YOLO exhibits fewer missed detections and more precise localization compared to the baseline. The improved performance is attributed to FCM’s enhanced spatial information propagation, which helps distinguish individual spikes in densely packed areas.

#### 4.7.2 Small spike detection

For small wheat spikes that are easily overlooked in deep feature extraction, LiteMS-YOLO demonstrates superior detection capability. The MKP unit’s multi-scale perception enables the network to capture fine-grained features essential for small target recognition.

#### 4.7.3 Complex backgrounds

In images with complex backgrounds—including varying illumination, soil exposure, and weed interference LiteMS-YOLO maintains robust detection performance. The complementary mapping mechanism helps the network focus on relevant spike features while suppressing background distractions.

**Figure 4:**
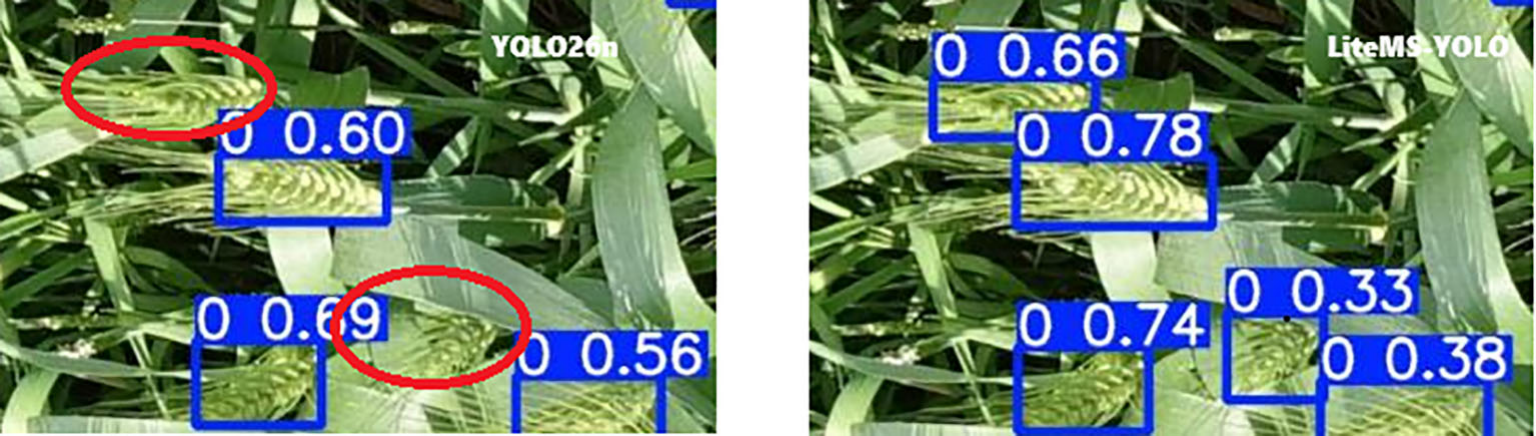
Side-by-side comparison of YOLO26n vs. LiteMS-YOLO in complex backgrounds (red circles indicate missed spikes by YOLO26n).

In complex wheat field environments, small target detection remains a critical challenge. As shown in the **Figure 4**, YOLO26n fails to detect several wheat spikes (highlighted by red circles) due to limited spatial information preservation and inadequate multi-scale perception. In contrast, LiteMS-YOLO (right column) successfully identifies these missed spikes, benefiting from the Feature Complementary Mapping (FCM) module and the Multi-Kernel Perception (MKP) unit. These results qualitatively validate the proposed method’s advantage in reducing false negatives and improving detection robustness under background clutter, occlusion, and scale variation.

The last two rows **Figure 3** displays feature map heatmaps comparing baseline YOLO26n and LiteMS-YOLO. The heatmaps reveal that LiteMS-YOLO exhibits stronger activation on wheat spike regions, with sharper focus and reduced background noise. This qualitative evidence supports the quantitative results, demonstrating that the proposed modules enhance the network’s ability to attend to relevant features.

### 4.8 Efficiency analysis

The efficiency score of LiteMS-YOLO reaches 147.17, which is approximately 4.02× higher than that of YOLO26n (36.16), demonstrating its superior parameter efficiency. This confirms that the integration of FCM, MKP, and redundancy reduction effectively improves model efficiency.

### 4.9 Training curve analysis

**Figure 5:**
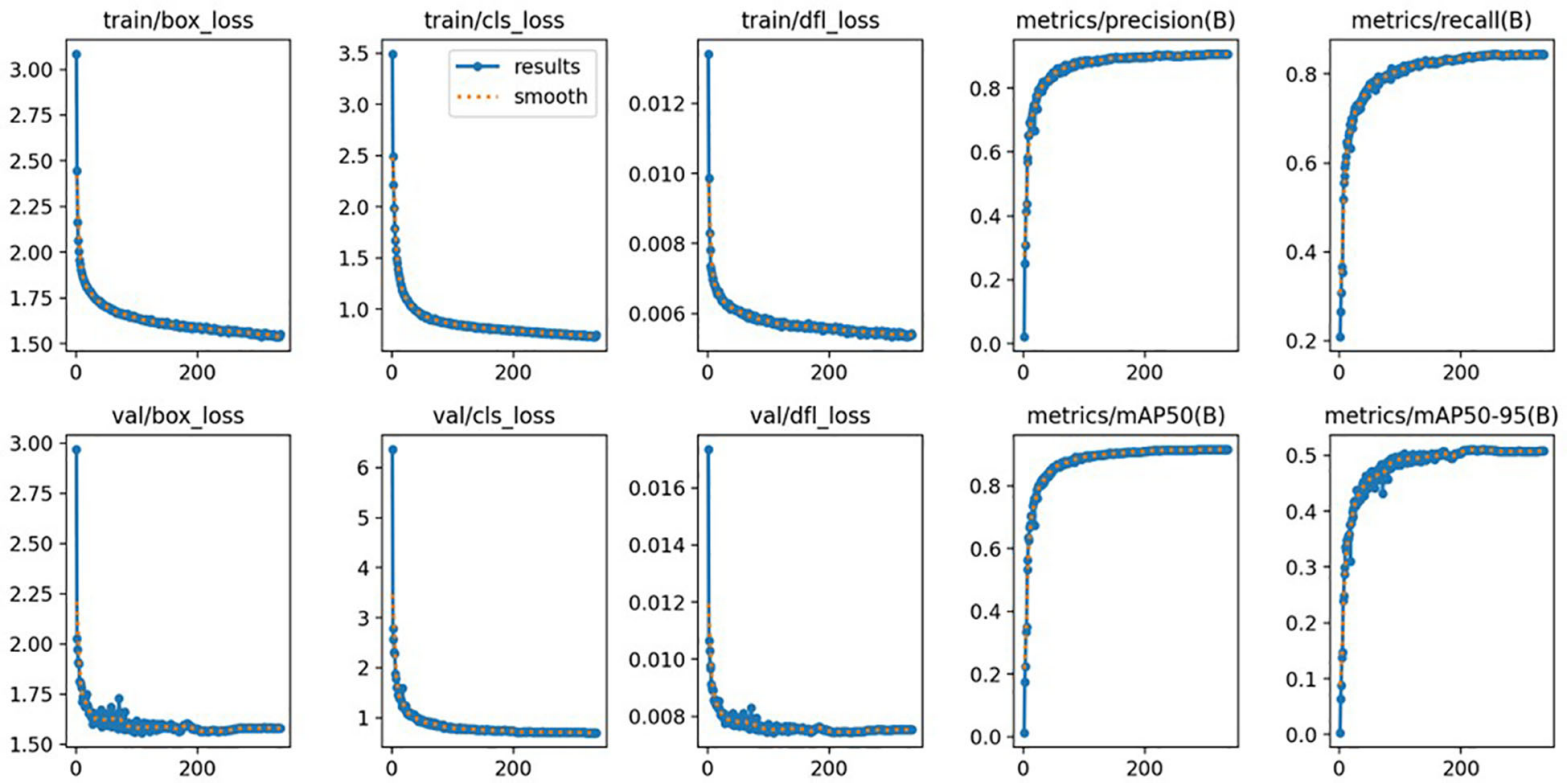
Training curves of LiteMS-YOLO over 300 epochs. Top row: training losses (box loss, classification loss, DFL loss). Bottom row: validation metrics (Precision, Recall, mAP@0.5, mAP@0.5:0.95).

To further evaluate the training dynamics of LiteMS-YOLO, we analyze the training curves recorded during model optimization. **Figure 5** illustrates the training metrics of LiteMS-YOLO over 300 epochs, including box loss, classification loss, distribution focal loss, as well as validation metrics including Precision, Recall, mAP@0.5, and mAP@0.5:0.95. The curves demonstrate that LiteMS-YOLO converges stably and smoothly throughout the training process. Both training and validation losses decrease consistently, indicating effective learning without overfitting. The mAP@0.5 on the validation set reaches a plateau near epoch 280, confirming the model’s robustness and stability.

### 4.10 Confusion matrix analysis

**Figure 6:**
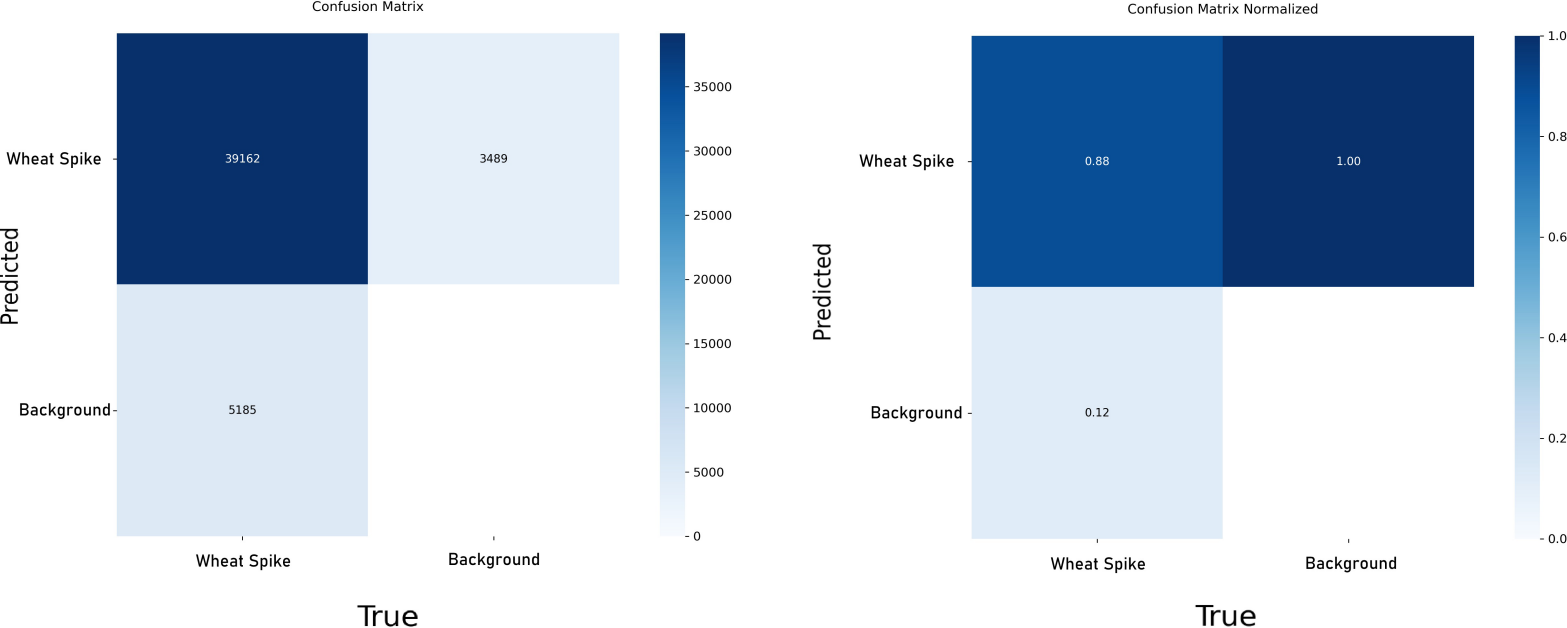
Confusion matrix analysis of LiteMS-YOLO on wheat spike detection dataset (Left: non-normalized confusion matrix, Right: normalized confusion matrix).

The normalized confusion matrix of LiteMS-YOLO is shown in **Figure 6**, and the non-normalized confusion matrix is shown in Figure (confusion_matrix.png). Together, they reveal the classification behavior of the model. Since the detection task involves only a single class (wheat spike), the matrices primarily reflect the relationship between true positives and background false positives. The results show that LiteMS-YOLO achieves a high true positive rate for the wheat spike class, with relatively low background confusion. Specifically, the model correctly predicted 39,162 wheat spike instances, with 3,489 false positives (background misdetected as wheat spikes) and 5,185 false negatives (missed wheat spikes). This demonstrates that the FCM module effectively enhances spatial-semantic alignment, enabling the model to accurately distinguish wheat spikes from complex field backgrounds, including soil, weeds, and varying illumination conditions.

### 4.11 Precision-Recall curve

**Figure 7:**
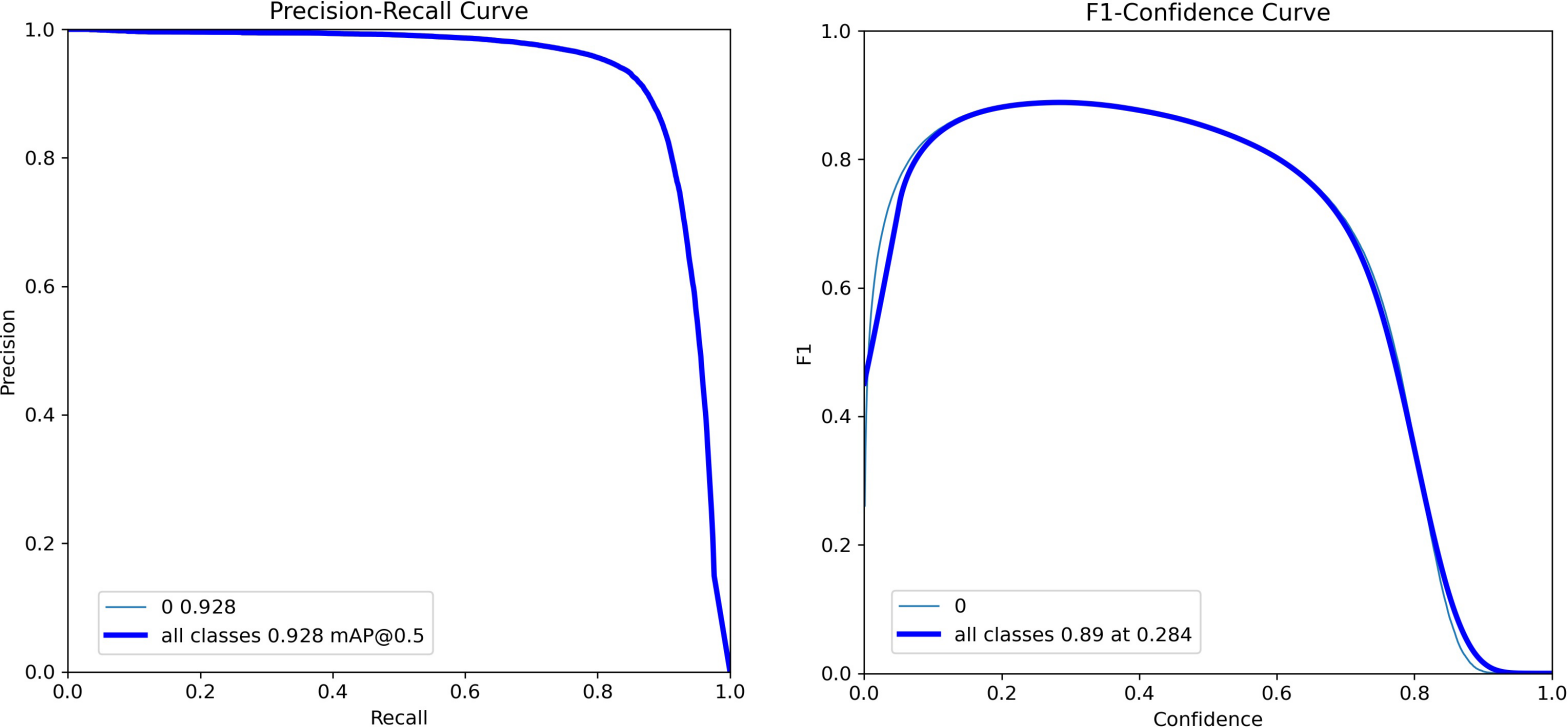
Precision-recall curve (left) and F1-score curve (right) of LiteMS-YOLO on the validation set.

**Figure 7** presents the Precision-Recall (PR) curve and F1-score curve of LiteMS-YOLO on the validation set. The PR curve demonstrates the model’s ability to maintain high precision across a wide range of recall values. The area under the PR curve (AP@0.5) reaches 0.928, consistent with the quantitative results reported in **Table 1**. The F1-score curve (**Figure 7**, right) shows that LiteMS-YOLO achieves a peak F1 score above 0.89 at a confidence threshold near 0.4, indicating a well-balanced trade-off between precision and recall. These results further validate the effectiveness of the proposed lightweight architecture in accurately detecting wheat spikes under challenging real-world field conditions.

### 4.12 Limitations

Despite its strong performance, LiteMS-YOLO has certain limitations. The model’s detection accuracy, while competitive, does not surpass the larger models (e.g., YOLOv8n) that utilize significantly more parameters and computational resources. There remains a trade-off between extreme lightweight design and maximum detection accuracy.

## 5 Field deployment on DJI T100s UAV platform

### 5.1 Field deployment and data acquisition on UAV platform

**Figure 8:**
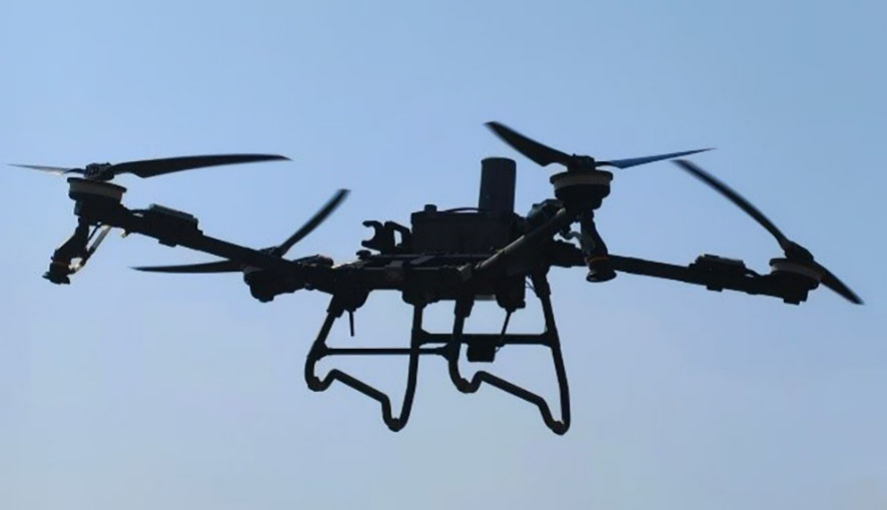
DJI T100s flying in the field.

**Figure 9:**
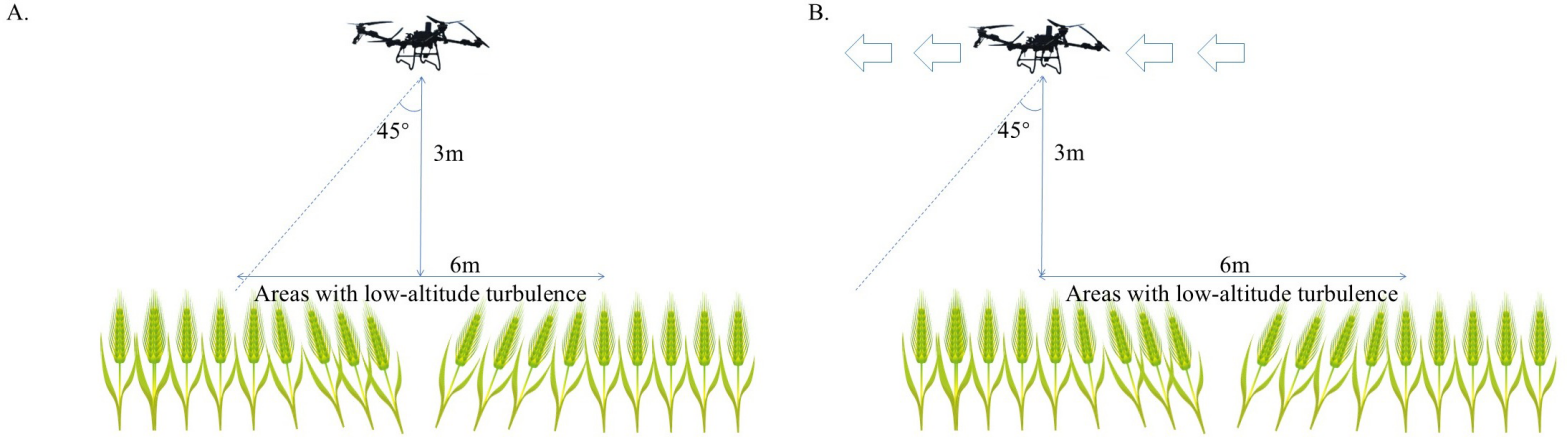
Schematic diagram of the UAV shooting geometry and turbulence zone: **(A)** UAV hovering, **(B)** UAV flying forward at 5 m/s.

To validate the practical effectiveness of LiteMS-YOLO, we conducted field experiments using the DJI T100s agricultural drone platform (**Figure 8**). This large-scale UAV generates strong downwash turbulence at 3 m altitude, causing rapid wheat spike oscillation. To obtain sharp images, we enforced a fixed shutter speed of 1/2000 s, a 0.5 s hover pre−stabilization, and 3−frame burst shooting per waypoint. The DJI Zenmuse P1 camera (45 MP, full-frame, 35 mm lens) was mounted at a 45° oblique angle (**Figure 9**), yielding an average camera−to−spike distance of 4.2 m. Quantitative evaluation confirmed that 95% of acquired images met the sharpness requirement for reliable small−target detection.

The field deployment experiments were conducted in a wheat growing region in Tangshan, Hebei Province, China, during the wheat heading stage.

### 5.2 Field dataset characteristics

A total of 2,847 field images were collected during the deployment experiments, covering approximately 15 hectares of wheat fields. The dataset exhibits significant diversity in terms of wheat growth stages, plant densities, and environmental conditions. The wheat spike targets in the field images vary considerably in scale due to different viewing angles and flight heights. Manual annotation was performed by agricultural experts, resulting in 48,326 labeled wheat spike instances across the dataset.

The dataset was split into training (70%), validation (15%), and test (15%) sets, ensuring that images from the same field location were not divided across different sets to avoid data leakage. The test set contains 427 images with 7,249 annotated wheat spikes, which were used for final performance evaluation.

### 5.3 Detection performance on field data

The detection performance of LiteMS-YOLO on the DJI T100s field dataset is summarized in **Table 5**. The model achieved an mAP@0.5 of 89.67% and an mAP@0.5:0.95 of 48.32% on the field test set, demonstrating robust performance under real-world conditions. The slightly lower performance compared to the GWHD dataset can be attributed to the challenging field environment, including occlusions from overlapping wheat leaves, varying illumination conditions, and the presence of foreground grass and background vegetation.

**Table 5:** Detection performance of LiteMS-YOLO on DJI T100s UAV field dataset.

Metric	Precision (%)	Recall (%)	mAP@0.5 (%)	mAP@0.5:0.95 (%)
LiteMS-YOLO	87.45	84.21	89.67	48.32

The Precision reached 87.45% and Recall reached 84.21%, indicating a well-balanced detection capability with minimal false positives and false negatives. The model successfully detected wheat spikes across various density levels, from sparse individual spikes to dense clusters with significant overlap.

### 5.4 Real-time performance analysis

**Table 6** presents the real-time inference performance of LiteMS-YOLO on the DJI T100s edge computing platform. The model processes input images at an average speed of 45.3 FPS with an average inference time of 22.1 milliseconds per frame. This performance enables real-time wheat spike detection during UAV flight operations, where the T100 drone maintains a ground speed of approximately 5 m/s. The effective ground coverage rate reaches 22.65 square meters per second, making it suitable for large-scale field surveying missions.

**Table 6:** Real-time performance on DJI T100s edge platform.

Metric	FPS	Inference time (ms)	Coverage rate (m2/s)
Value	45.3	22.1	22.65

### 5.5 Robustness analysis under field conditions

The detection performance under various field conditions is analyzed in **Table 7**. Under sunny conditions with strong direct sunlight, the model achieved an mAP@0.5 of 91.23%, demonstrating excellent robustness to bright illumination. Under cloudy conditions with diffused light, the mAP@0.5 reached 90.15%, showing slightly reduced performance due to lower image contrast. Under partially shaded conditions with mixed lighting, the performance decreased to 87.42%, primarily due to uneven illumination causing some wheat spikes to be underexposed or overexposed.

**Table 7:** Robustness analysis under different field lighting conditions.

Condition	Precision (%)	Recall (%)	mAP@0.5 (%)
Sunny	89.56	86.89	91.23
Cloudy	87.92	85.67	90.15
Partially Shaded	85.03	80.45	87.42

These results validate that LiteMS-YOLO maintains robust detection performance across diverse field conditions, making it highly suitable for practical agricultural deployment. The lightweight model architecture ensures that the detection system can operate effectively on UAV-mounted edge computing devices while maintaining accuracy comparable to cloud-based solutions.

## 6 Discussion

### 6.1 Key findings

The experimental results yield several key findings:

Lightweight design is achievable without sacrificing accuracy. LiteMS-YOLO demonstrates that careful architectural design can dramatically reduce model size (~75.0% reduction from baseline YOLO26n) while achieving state-of-the-art detection performance (mAP50: 92.28%, mAP50-95: 52.56%) in wheat spike detection. This finding has significant implications for deploying detection systems on edge devices in agricultural settings.

Information imbalance is a critical bottleneck. The FCM module’s consistent improvements across experiments validate the hypothesis that insufficient integration between shallow spatial features and deep semantic representations limits small object detection performance. Addressing this imbalance yields measurable accuracy gains.

Multi-scale perception benefits from progressive receptive field expansion. The MKP unit’s sequential application of varying kernel sizes with point-wise convolutions outperforms single-size or parallel multi-branch designs, suggesting that progressive feature refinement across scales captures complementary information effectively.

Synergistic integration amplifies benefits. The combination of FCM and MKP with targeted redundancy reduction produces the best overall results, demonstrating that these components address complementary aspects of the detection problem.

### 6.2 Design implications

The success of LiteMS-YOLO suggests several design principles for agricultural detection tasks:

#### 6.2.1 Prioritize spatial information preservation

Small target detection benefits significantly from preserving spatial information throughout the network. The FCM module’s approach of propagating shallow spatial features into deeper layers provides a template for addressing this need without substantial parameter overhead.

#### 6.2.2 Embrace multi-scale feature extraction

Agricultural targets often exhibit significant scale variations. Lightweight multi-scale modules like MKP that efficiently expand receptive fields offer better trade-offs than simply increasing network depth or width.

#### 6.2.3 Target redundancy elimination

Many baseline models contain architectural redundancies that can be eliminated with careful design. Decoupling downsampling from channel expansion, as demonstrated in this work, can substantially reduce parameters while maintaining capability.

### 6.3 Limitations and future work

Although LiteMS-YOLO achieves a favorable balance between lightweight characteristics and detection accuracy for wheat spikes in complex field environments, it still exhibits noticeable performance degradation under three challenging scenarios: extreme occlusion, ultra-small targets, and wheat spikes at different growth stages. These limitations stem from inherent structural constraints introduced by the extreme lightweight design and targeted module configurations, rather than general drawbacks of small-model deployment.

#### 6.3.1 Performance degradation under extreme occlusion

The detection accuracy drops significantly under heavy occlusion primarily due to insufficient global context modeling and restricted receptive fields. The depth-aware FCM module prioritizes spatial–semantic alignment for dense small targets but weakens long-range dependency capture, making the model unable to infer complete spike structures from partial visible regions. The sequential MKP unit adopts fixed small-to-medium convolution kernels (3×3, 5×5, 7×7) to ensure computational efficiency, which limits the receptive field scale and impairs the perception of occluded targets. In addition, the targeted redundancy reduction strategy compresses channel dimensions, reducing the representation capacity of local fragmentary features that are critical for occluded spike recognition.

Performance degradation for ultra-small wheat spikes For ultra-small wheat spikes (usually less than 10×10 pixels), repeated downsampling operations in the lightweight backbone further shrink effective spatial resolution, leading to irreversible loss of fine-grained texture and edge features. Although the FCM module alleviates spatial information decay in deep layers, the severely reduced channel capacity after redundancy reduction weakens the feature expression for tiny targets. Furthermore, the multi-scale detection head is designed for general wheat spike sizes and lacks a dedicated prediction branch and enhanced feature allocation for ultra-small objects, resulting in low recall for extremely tiny targets.

Performance fluctuation across different growth stages Wheat spikes exhibit substantial variations in morphology, size, color, and texture across different growth stages (e.g., booting, heading, flowering, and filling stages). The shallow network depth and limited feature diversity caused by lightweight optimization restrict the model’s ability to adapt to large intra-class appearance changes. The fixed multi-kernel configuration in MKP cannot dynamically adjust receptive fields or feature weights according to growth-stage variations. Meanwhile, the absence of growth-stage-adaptive feature normalization or augmentation makes the model sensitive to phenotypic changes rather than focusing on invariant spike characteristics, leading to unstable detection across phenological phases.

To overcome the aforementioned limitations, we propose four targeted improvement directions for subsequent research:

Introduce lightweight transformer-based global attention to compensate for insufficient long-range context modeling, enhancing the detection robustness for heavily occluded and scattered targets.

Incorporate feature super-resolution enhancement modules to recover lost spatial details in deep layers, improving the representation and recall of ultra-small wheat spikes.

Design dynamic receptive field adjustment or growth-stage-aware adaptive detection heads to enable adaptive feature perception for wheat spikes at different phenological phases, reducing performance fluctuations.

Fuse multispectral or hyperspectral imaging data to provide complementary phenotypic information beyond RGB features, further improving detection stability under occlusion, illumination variations, and growth-stage changes.

Extensive validation will be conducted on wheat datasets from more geographical regions, varieties, and planting patterns to further verify and improve the cross-scenario generalization ability of the model.

## 7 Conclusion

This paper presented LiteMS-YOLO, a novel lightweight detection framework for accurate wheat spike detection in complex field environments. The proposed method addresses two fundamental challenges in small object detection: the information imbalance between shallow spatial features and deep semantic representations, and the need for efficient multi-scale feature extraction. The Feature Complementary Mapping (FCM) module effectively propagates spatial information into deeper network layers through a split-transform-aggregation strategy, enhancing feature alignment for small target localization. The Multi-Kernel Perception (MKP) unit leverages sequential depthwise convolutions with varying kernel sizes interleaved with point-wise convolutions to capture multi-scale features while maintaining computational efficiency.

Comprehensive experiments on the GWHD dataset demonstrate that LiteMS-YOLO achieves a mAP50 of 92.28% and a mAP50–95 of 52.56% on the GWHD dataset, while using only 0.627 million parameters, representing a reduction of approximately 75% compared to the baseline YOLO26n. Ablation studies validate the effectiveness of each component, and qualitative results show improved detection in dense spike scenarios, small target regions, and complex backgrounds.

The exceptional parameter efficiency of LiteMS-YOLO makes it highly suitable for deployment on resource-constrained edge devices, enabling real-time wheat spike detection in agricultural field settings. This work contributes to the advancement of precision agriculture by providing an efficient and accurate solution for yield estimation and phenotypic analysis. The code and models will be made publicly available to facilitate further research in this important domain.

## Data Availability

The original contributions presented in the study are included in the article/supplementary material. Further inquiries can be directed to the corresponding authors.

## References

[B1] Bochkovskiy A. Wang C.-Y. Liao H.-Y. M. (2020). YOLOv4: optimal speed and accuracy of object detection. doi: 10.48550/arxiv.2004.10934

[B2] Cheng A. Xiao J. Li Y. Sun Y. Ren Y. Liu J. (2024). Enhancing remote sensing object detection with K-CBST YOLO: integrating CBAM and Swin-Transformer. Remote Sens. 16, 2885. doi: 10.3390/rs16162885 30654563

[B3] David E. Madec S. Sadeghi-Tehran P. Aasen H. Zheng B. Liu S. . (2020). Global Wheat Head Detection (GWHD) dataset: a large and diverse dataset of high-resolution RGB-labelled images to develop and benchmark wheat head detection methods. Plant Phenomics 2020, 3521852. doi: 10.34133/2020/3521852 33313551 PMC7706323

[B4] Fang C. Yang X. (2024). Lightweight YOLOv8 for wheat head detection. IEEE Access 12, 66214–66222. doi: 10.1109/access.2024.3397556 25079929

[B5] Guan S. Lin Y. Lin G. Su P. Huang S. Meng X. . (2024). Real-time detection and counting of wheat spikes based on improved YOLOv10. Agronomy 14, 1936. doi: 10.3390/agronomy14091936 30654563

[B6] Hasan M. M. Chopin J. P. Laga H. Miklavcic S. J. (2018). Detection and analysis of wheat spikes using convolutional neural networks. Plant Methods 14, 100. doi: 10.1186/s13007-018-0366-8 30459822 PMC6236889

[B7] Hidayatullah P. Tubagus R. (2026). YOLO26: a comprehensive architecture overview and key improvements. doi: 10.48550/arxiv.2602.14582

[B8] Hoanh N. Pham T. V. (2024). Focus-attention approach in optimizing DETR for object detection from high-resolution images. Knowledge-Based Syst. 296, 111939. doi: 10.1016/j.knosys.2024.111939 38826717

[B9] Khazmi K. Lachiri Z. (2024). “ A comparative study for wheat head detection through testing the robustness of two global dataset trained YOLO models on a Tunisian wheat dataset”, in: 2024 IEEE International Conference on Artificial Intelligence & Green Energy (ICAIGE) (Yasmine Hammamet, Tunisia: IEEE), 1–6. doi: 10.1109/icaige62696.2024.10776750

[B10] Kong Y. Shang X. Jia S. (2024). Drone-DETR: efficient small object detection for remote sensing image using enhanced RT-DETR model. Sensors 24, 5496. doi: 10.3390/s24175496 39275406 PMC11397902

[B11] Lei M. Li S. Wu Y. Hu H. Zhou Y. Zheng X. . (2025). YOLOv13: real-time object detection with hypergraph-enhanced adaptive visual perception. doi: 10.48550/arxiv.2506.17733

[B12] Li X. Cai M. Tan X. Yin C. Chen W. Liu Z. . (2024). An efficient transformer network for detecting multi-scale chicken in complex free-range farming environments via improved RT-DETR. Comput. Electron. Agric. 224, 109160. doi: 10.1016/j.compag.2024.109160 38826717

[B13] Liang Z. Xu X. Yang D. Liu Y. (2025). The development of a lightweight DE-YOLO model for detecting impurities and broken rice grains. Agriculture 15, 848. doi: 10.3390/agriculture15080848 30654563

[B14] Lin T.-Y. Dollar P. Girshick R. He K. Hariharan B. Belongie S. (2017). “ Feature pyramid networks for object detection”, in: 2017 IEEE Conference on Computer Vision and Pattern Recognition (CVPR) (Honolulu, HI, USA: IEEE), 936–944. doi: 10.1109/cvpr.2017.106

[B15] Liu C. Guo J. Yang Y. (2026). Research on field wheat maturity detection algorithm based on improved faster R-CNN. Eng. Res. Express 8, 015514. doi: 10.1088/2631-8695/ae310e

[B16] Liu C. Zhang S. Hu M. Song Q. (2024). Object detection in remote sensing images based on adaptive multi-scale feature fusion method. Remote Sens. 16, 907. doi: 10.3390/rs16050907 30654563

[B17] Liu S. Qi L. Qin H. Shi J. Jia J. (2018). “ Path aggregation network for instance segmentation”, in: 2018 IEEE/CVF Conference on Computer Vision and Pattern Recognition (Salt Lake City, UT, USA: IEEE), 8759–8768. doi: 10.1109/cvpr.2018.00913

[B18] Qiu Z. Wang F. Li T. Liu C. Jin X. Qing S. . (2025). LGWheatNet: a lightweight wheat spike detection model based on multi-scale information fusion. Plants 14, 1098. doi: 10.3390/plants14071098 40219167 PMC11991583

[B19] Redmon J. Divvala S. Girshick R. Farhadi A. (2016). “ You only look once: unified, real-time object detection”, in: 2016 IEEE Conference on Computer Vision and Pattern Recognition (CVPR) (Las Vegas, NV, USA: IEEE), 779–788. doi: 10.1109/cvpr.2016.91

[B20] Ren S. He K. Girshick R. Sun J. (2017). Faster R-CNN: towards real-time object detection with region proposal networks. IEEE Trans. Pattern Anal. Mach. Intell. 39, 1137–1149. doi: 10.1109/tpami.2016.2577031 27295650

[B21] Terven J. Cordova-Esparza D. (2023). A comprehensive review of YOLO architectures in computer vision: from YOLOv1 to YOLOv8 and YOLO-NAS. doi: 10.48550/arxiv.2304.00501

[B22] Wang K. Hu X. Zheng H. Lan M. Liu C. Liu Y. . (2024). Weed detection and recognition in complex wheat fields based on an improved YOLOv7. Front. Plant Sci. 15, 1372237. doi: 10.3389/fpls.2024.1372237 38978522 PMC11228305

[B23] Wang X. Li C. Zhao C. Jiao Y. Xiang H. Wu X. . (2025b). GrainNet: efficient detection and counting of wheat grains based on an improved YOLOv7 modeling. Plant Methods 21, 44. doi: 10.1186/s13007-025-01363-y 40128728 PMC11934480

[B24] Wang D. Shi L. Yin H. Cheng Y. Liu S. Wu S. . (2025a). A detection approach for wheat spike recognition and counting based on UAV images and improved faster R-CNN. Plants 14, 2475. doi: 10.3390/plants14162475 40872097 PMC12389362

[B25] Wang Q. Wu B. Zhu P. Li P. Zuo W. Hu Q. (2020). “ ECA-Net: efficient channel attention for deep convolutional neural networks”, in: 2020 IEEE/CVF Conference on Computer Vision and Pattern Recognition (CVPR) (Seattle, WA, USA: IEEE), 11531–11539. doi: 10.1109/cvpr42600.2020.01155

[B26] Wen C. Ma Z. Ren J. Zhang T. Zhang L. Chen H. . (2024). A generalized model for accurate wheat spike detection and counting in complex scenarios. Sci. Rep. 14, 24189. doi: 10.1038/s41598-024-75523-w 39407029 PMC11480395

[B27] Wu H. Wu W. Huang Y. Liu S. Liu Y. Zhang N. . (2025). FEWheat-YOLO: a lightweight improved algorithm for wheat spike detection. Plants 14, 3058. doi: 10.3390/plants14193058 41095199 PMC12526082

[B28] Xiao Y. Xu T. Xin Y. Li J. (2025). “ FBRT-YOLO: faster and better for real-time aerial image detection”, in: Proceedings of the AAAI Conference on Artificial Intelligence (Washington, DC: AAAI Press), 39, 8673–8681. doi: 10.1609/aaai.v39i8.32937

[B29] Xiong H. Cao Z. Lu H. Madec S. Liu L. Shen C. (2019). TasselNetv2: in-field counting of wheat spikes with context-augmented local regression networks. Plant Methods 15, 150. doi: 10.1186/s13007-019-0537-2 31857821 PMC6905110

[B30] Yang L. Yan J. Li H. Cao X. Ge B. Qi Z. . (2022). Real-time classification of invasive plant seeds based on improved YOLOv5 with attention mechanism. Diversity 14, 254. doi: 10.3390/d14040254 30654563

[B31] Zang H. Wang Y. Ru L. Zhou M. Chen D. Zhao Q. . (2022). Detection method of wheat spike improved YOLOv5s based on the attention mechanism. Front. Plant Sci. 13, 993244. doi: 10.3389/fpls.2022.993244 36247573 PMC9554473

[B32] Zhang Y. Liu Z. Guo X. Li C. Teng G. (2025). Wheat head detection in field environments based on an improved YOLOv11 model. Agriculture 15, 1765. doi: 10.3390/agriculture15161765 30654563

[B33] Zhaosheng Y. Tao L. Tianle Y. Chengxin J. Chengming S. (2022). Rapid detection of wheat ears in orthophotos from unmanned aerial vehicles in fields based on YOLOX. Front. Plant Sci. 13, 851245. doi: 10.3389/fpls.2022.851245 35574098 PMC9094485

[B34] Zou Y. Tian Z. Cao J. Ren Y. Zhang Y. Liu L. . (2023). Rice grain detection and counting method based on TCLE–YOLO model. Sensors 23, 9129. doi: 10.3390/s23229129 38005517 PMC10675024

